# Unexpected Giant-Gap Quantum Spin Hall Insulator in Chemically Decorated Plumbene Monolayer

**DOI:** 10.1038/srep20152

**Published:** 2016-02-02

**Authors:** Hui Zhao, Chang-wen Zhang, Wei-xiao Ji, Run-wu Zhang, Sheng-shi Li, Shi-shen Yan, Bao-min Zhang, Ping Li, Pei-ji Wang

**Affiliations:** 1 School of Physics and Technology, University of Jinan, Jinan, Shandong, 250022, People’s Republic of China; 2 School of Physics, State Key laboratory of Crystal Materials, Shandong University, Jinan, Shandong, 250100, People’s Republic of China

## Abstract

Quantum spin Hall (QSH) effect of two-dimensional (2D) materials features edge states that are topologically protected from backscattering by time-reversal symmetry. However, the major obstacles to the application for QSH effect are the lack of suitable QSH insulators with a large bulk gap. Here, we predict a novel class of 2D QSH insulators in X-decorated plumbene monolayers (PbX; X = H, F, Cl, Br, I) with extraordinarily giant bulk gaps from 1.03 eV to a record value of 1.34 eV. The topological characteristic of PbX mainly originates from *s-p*_*x,y*_ band inversion related to the lattice symmetry, while the effect of spin-orbital coupling (SOC) is only to open up a giant gap. Their QSH states are identified by nontrivial topological invariant Z_2_ = 1, as well as a single pair of topologically protected helical edge states locating inside the bulk gap. Noticeably, the QSH gaps of PbX are tunable and robust *via* external strain. We also propose high-dielectric-constant BN as an ideal substrate for the experimental realization of PbX, maintaining its nontrivial topology. These novel QSH insulators with giant gaps are a promising platform to enrich topological phenomena and expand potential applications at high temperature.

Topological insulators (TIs)^1^,^2^,^3^,^4^, as a new class of quantum materials, have generated intensive research activities in quantum information, because they give an alternative and robust platform for obtaining relativistic and spin-polarized Fermions in the condensed matter system. One of the most interesting phenomena in this realm is the quantum spin Hall (QSH) effect in two-dimensional (2D) materials^1^,^2^, characterized by an insulating bulk-gap and gapless edge states at its boundaries due to time-reversal symmetry (TRS), thus providing the enticing concepts for novel quantum electronic devices with low energy dissipation^3^,^4^. The prototypical concept of QSH insulator is first proposed by Kane and Mele in graphene^5^,^6^, in which the spin-orbit coupling (SOC) opens a band gap at the Dirac point. However, the associated gap due to rather weak second-order effective SOC is too small (~10^−3^ meV), which makes the QSH state in graphene only appear at an unrealistically low temperature. Quantized conductance through QSH edge states have only been experimentally demonstrated in HgTe/CdTe^7^,^8^ and InAs/GaSb^9^,^10^ quantum wells at the ultralow temperature, which greatly obstructs their potential applications in spintronics.

To be “good” QSH insulators, the materials should have a large bulk-gap to realize the spin-polarized electron transport at room temperature. Controlling the chemical bonding at atomic levels to induce the band inversion^3^,^4^ by SOC is an efficient way to realize topologically nontrivial Z_2_ number^8^,^11^,^12^,^13^,^14^,^15^. The 2D thin films are advantageous in this aspect as their bonding properties are easy to be modified in post synthesis processes, for example, by surface adsorption to enlarge SOC strength. Group-IV honeycomb lattices such as hydrogenated or halogenated germanene and stanene^16^,^17^ monolayers have been reported as a QSH phase with a bulk-gap as large as 0.2–0.3 eV. However, most QSH phases in these systems are not intrinsic, but driven by external strain, thus are unfavorable for practical applications in spintronics. On the other hand, Rivero *et al.*^18^ recently reported the stability and properties of high-buckled 2D Sn and Pb thin films. They find that the optimal structure of fluorinated stanene lacks threefold symmetry and thus the experimental realization of 2D fluorinated one is challengeable. The strong SOC effect can also be sufficed in group-V heavy elements such as bismuth, which drives a nontrivial QSH state^19^. Besides, an approach to design a large-gap QSH state on a semiconductor surface by a substrate orbital filtering process is also proposed^20^,^21^. More recently, the ethynyl-functionalized stanene has been reported to be a good QSH insulator^22^. These large-gap QSH insulators are essential for realizing many exotic phenomena and for fabricating new quantum devices that can operate at room temperature.

In this work, on the basis of first-principles calculations, we predicted a class of new QSH insulators in X-decorated plumbene monolayers (PbX; X = H, F, Cl, Br, and I), in analogy 2D hydrocarbon (graphane)^23^,^24^. An extraordinarily nontrivial giant-gap in the range of 1.03 ~ 1.34 eV were obtained, making the experimental observation of QSH phase facile. The topological characteristic of PbX was attributed to *s-p*_*x,y*_ band inversion related to the lattice symmetry, while the effect of SOC was only to open up a giant-gap. Interestingly, the QSH gaps of PbX were tunable and robust against external strain. Our findings had potential applications in low-power quantum electronics and may enable topological quantum computing based on Majorana fermions^25^.

Figure 1(a) shows the typical lattice structure of 2D PbX, in which the Pb atoms are in sp^3^ hybridization forming a hexagonal network, along with X being bonded to Pb atoms on both sides of the plane in an alternating manner. In comparison with the pristine plumbene in Fig. S1, all Pb-Pb bonds in PbX slightly expand, while the buckling decreases clearly due to the weakly hybridization between π and σ orbitals, as listed in Table 1. We also note the similar results in halogenated germanene and stanene monolayers^16^,^17^. The buckling plays a crucial role in the engineering of the band structures in these materials. The high structural stability is verified by the formation energy defined as


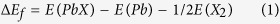


where *E*(PbX) and *E*(Pb) are the total energies of decorated and pristine plumbene, respectively, while *E*(X_2_) is the chemical potential of hydrogen or halogen molecules. The calculated formation energy *ΔE* is found to be −1.89, −2.82, −1.48, −1.32, and −0.86 eV/atom for PbH, PbF, PbCl, PbBr, and PbI, respectively, indicating no phase separation in these systems. Besides, we also calculate the phonon dispersion for PbX monolayers, as illustrated in Fig. 1(c) and Fig. S2. The frequencies of all modes are positive over the whole Brillouin zone, implying that PbX are thermodynamically stable.

We now focus on the band structures of PbH monolayer as an example. In the absence of SOC (Fig. 2(a)), the band gap at the K point is substantially enlarged due to the saturation of the Pb-*p*_*z*_, maintaining *p*_*x,y*_ level located at the Γ point. Thus, PbH can be regarded as a gapless semiconductor. By further projecting the bands onto different atomic orbitals, we find that the energy spectrums at the Γ point mainly come from one *s* and two *p* orbitals of Pb atoms. When the effect of SOC is taken into consideration (Fig. 2(b)), the energy degeneracy of *p*_*x,y*_ orbitals at the Γ point are lifted, indicating a semimetal-to-semiconductor transition. Away from the Г point, the conduction bands are upshifted, whereas the valence bands are downshifted, which produces a global indirect-gap as large as 0.98 eV, ample for applications at room temperature. These suggest that PbH could be TIs upon opening a band-gap at the touching point by SOC. To further confirm these results, we also have employed HSE06^26^ to check the electronic structures of PbH monolayer in Fig. S3, which are in good agreement with these obtained here.

The SOC-induced gap opening near the Fermi level indicates possible existence of 2D TI phase that are helical with the spin-momentum locked by TRS. Thus, we calculate the topological edge states of PbH by the Wannier90 package^27^. We construct the maximally localized Wannier functions (MLWFs) and fit a tight-binding Hamiltonian with these functions. Figure 3(a) shows the DFT and fitted band structures, in well agreement with each other. Then, the edge Green’s function^28^ of a semi-infinite PbH is constructed and the local density of state (LDOS) of PbH is calculated, as shown in Fig. 3(c). Clearly, all the edge bands connect completely the conduction and valence bands and span the 2D bulk band gap, yielding 1D gapless edge states. Besides, the counter-propagating edge states exhibit opposite spin polarizations, in accordance with the spin-momentum locking of 1D helical electrons (Fig. 3(d)). On the other hand, we construct a zigzag-edged nanoribbon, as shown in Fig. 3(e). All the edge Pb atoms are hydrogenated to eliminate the angling bonds. The width of the nanoribbon, 20.6 nm, is large enough to avoid interactions between the edge states of the two sides, and the band structure of the nanoribbon is shown in Fig. 3(f). We can explicitly observe that the gapless edge states (red lines) that appear within the bulk gap and cross linearly at the Γ point, demonstrating the topological nontribal property of the bulk gap, which is consistent with the Fig. 3(b). In this case, the Dirac point located at the band gap is calculated to have a high velocity of ~1.0 × 10^5^ m/s, larger than that of 3.0 × 10^4^ m/s in InAs/GaSb quantum well^9^,^10^, indicating that PbH is an ideal 2D TI.

To further confirm the topological edge states of PbH monolayer, we calculate the Z_2_ invariant *v* based on the method proposed by Fu and Kane^29^, due to the presence of structural inversion symmetry. Here, the topological index ν can be established by


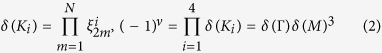


Where *δ* is the product of parity eigenvalues at the time-reversal-invariant momenta (TRIM) points, *ξ* = ±1 are the parity eigenvalues and *N* is the number of the occupied bands. According to the Z_2_ classification, ν = 1 characterizes a topologically nontrivial phase and ν = 0 means a topologically trivial one. Here, the invariants Z_2_ are derived from the parities of wave function at the four TRIM points K_i_, including Γ(0, 0), *M*_1_(0, 1/2), *M*_2_(1/2, 0), and *M*_3_(1/2, 1/2), as illustrated in Fig. 1(d). From the calculated results in Fig. 2(c), we find the Z_2_ of PbH monolayer is +1, indicating that it is a QSH insulator.

To understand the physical origin of QSH state, we next do an orbital analysis around the Fermi level for PbH monolayer. As can be seen from density of states (DOS) in Fig. 3(c), the decorated H atoms hybridizes strongly with the dangling bonds of *p*_*z*_ orbital in Pb atoms overlapping in the same energy range, which effectively removes the *p*_*z*_ bands away from the Fermi level, leaving only the *s* and *p*_*x,y*_ states at the Fermi level. Thus, we present systematically the band evolution at the Γ point for PbH monolayer in Fig. 4(a). One can see that the chemical bonding and crystal field splitting between Pb-Pb atoms make the *s* and *p*_*x,y*_ orbitals split into the bonding and anti-bonding states, i.e., 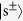 and 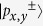, which the superscripts + and − represent the parities of corresponding states, respectively. Noticeably, the band orders of PbH are different from the previous reported cases in GeH/SnH^16^,^17^, where the 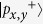 lies below 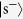 orbital, which leads to inversion of the bands around the Fermi level, to produce the crucial QSH effect. This is because that the modification of band order upon surface functionalization is related to the bond length and orbital splitting. The larger lattice constant of PbH monolayer (4.978 Å) results in a weaker *s-p* hybridization, and accordingly a smaller energy separation between the bonding and antibonding states. Thus, the 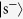 orbital is downshifted while the 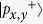 orbital is upshifted, i.e., the 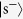 will be occu*p*ied, while the quadruply degenerate 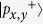 is half occupied (due to the C_3_ rotation symmetry), resulting in semi-metallic character (Fig. 2(a)). With the inclusion of SOC, the degeneracy of the level splits into 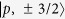 state with a total angular momentum *j* = 3/2 and 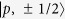 with a total angular momentum *j* = 1/2, opening a full band gap. Obviously, the adsorbed hydrogen atoms act like an orbital filter to selectively remove the *p*_*z*_ orbitals from the Pb lattice, maintaining a nontrivial six-band lattice with Z_2_ = 1^30^. Here, an interesting feature of band structure is that the effect of SOC is only producing an band gap around the Fermi level, but not inducing band inversion, i.e., SOC is not relevant for the formation of nontrivial band orders. The brand new mechanism here may provide an efficient way to search for large-gap QSH insulators in 2D materials^20^,^21^. In fact, a similar situation has even been observed in well-known 2D TIs such as graphene and silicene^5^,^6^,^12^, where the inclusion of SOC does not change the band order between π and π* bands. However, the nontrivial QSH effect of graphene mainly originates from the massive Dirac cone^5^,^6^, but the QSH phase in PbH is attributed to *s-p* band inversion at Г the point, similar to that in the Bi_2_Se_3_ or HgTe quantum well^8^,^13^.

Here, it is worth emphasizing that the hydrogenation in plumbene is not the only way to achieve the giant-gap QSH state, the same results can be obtained by decorating the surface with otherwise halogen atoms, such as F, Cl, Br, and I. We thus performed calculations for PbX (X = F, Cl, Br, I) to check their topological properties, as illustrated in Figs S3–S5. Table 1 summarizes their lattice constants, Pb-Pb bond lengths, and nontrivial gaps at their equilibrium states. These results demonstrate that all the electronic structures of halogenated PbX monolayers are similar to PbH, and exhibit nontrivial topological invariant Z_2_ = 1(Table S1). Noticeably, as can be seen in Fig. 4(c) and Fig. S2, the global QSH gaps of PbF, PbCl, PbBr, and PbI are 1.34, 1.19, 1.15, and 1.03 eV, respectively, which are sufficiently large for practical applications at high temperature. On the other hand, we can find some interesting phenomena when comparing the band gaps with each other. It is known that, from F to I, the SOC becomes stronger in the order of F < Cl < Br < I, thus the SOC induced band gap should follow this trend. Since the band gaps of PbX monolayers are mainly determined by SOC strength near the Fermi level, this order also works. However, the fact is just opposite, where the band gaps of PbX decrease monotonically, namely, PbF > PbCl > PbBr > PbI. Further explanation of this interesting contradiction can be attributed to the band components of Pb atoms near the Fermi level, as its splitting induced by SOC can directly determine QSH gap. From Fig. 4(d), one can see that the ratio from the Pb-*p*_*x,y*_ to bonded X orbital at the Γ point near the Fermi level decreases in the order of PbF > PbCl > PbBr > PbI. Considering that the Pb exhibits stronger SOC strength than X atoms, it is expected that the larger the ratio is, the larger the contribution to the orbital near the Fermi level, and consequently the larger the SOC strength will be. As a result, we can understand why the global band gaps of these systems decrease in the contrary order of PbF > PbCl > PbBr > PbI.

The buckle structure generally sustain a larger strain than planar one, thus one might ask if these QSH insulators are robust under mechanical strain? In what follows, we investigate the effect of biaxial strain on topological properties of PbX monolayers. Here, we employ an in-plane strain on PbX maintaining the crystal symmetry by changing its lattices as *ε* = (a − a_0_)/a_0_, where the a (a_0_) is strained (equilibrium) lattice constants. Figure 5 gives the variation of the direct gap (*E*_g_ (Γ)) at the Γ point and indirect gap (*E*_g_) with respect to external strain, which indicates the interatomic coupling can indeed modulate the topological properties. Especially, the QSH feature is rather robust when the strain falls into the range from −6 to +8%. Both the direct and indirect gaps decrease slightly with respect to tensile strain, but their nontrivial bulk-gaps within the strain range are still very large, i.e., larger than 0.96 eV of *E*_g_ for 8% strain for PbF monolayer. Such robust topology paves the easy way for experimental realization.

It is noticeable that, although the 2D QSH insulators have been extensively sought and studied theoretically and experimentally^10^,^11^,^12^,^13^,^14^,^15^,^16^,^17^,^18^,^19^,^20^,^21^, but few TIs with a bulk-gap exceeding 1.0 eV has been discovered up to now. Here, a giant-gap as large as 1.34 eV in PbF monolayer is the largest bulk-gap of all the reported 2D TIs, which are approximately four times the values of halogenated GeX/SnX films (~0.3 eV) and superstar 3D Bi_2_Se_3_ film (~0.35 eV)^13^. The topological states with a giant-gap are mainly due to the contribution of *p*_x_ and *p*_y_ orbitals of Pb atoms, instead of the next-nearest-neighbor SOC in the p_z_ states^5^,^6^. These giant bulk-gaps are advantageous for stabilizing the edge currents against the interference of thermally activated carriers in the bulk, which are beneficial to applications at room temperature.

On the experimental side, high-quality plumbene film in a honeycomb lattice has been successfully grown on different substrates based on the MBE method^31^. Also, a hydrogenated graphene and germanene^32^,^33^ are experimentally synthesized. Thus, it is expected that the PbX monolayers can also be fabricated or transferred on the substrate using similar techniques. As a 2D large-gap insulator with a high dielectric constant, BN sheet has been successfully used as the substrate to grow graphene or assembled in 2D stacked nanodevices^34^,^35^. Thus, we select the BN-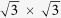
(4.53 Å) and BN-2 × 2 (5.23 Å) as the substrate to support PbH monolayer. The substrate is modeled by placing 1–5 BN layers under PbH where all atoms in the bottom BN layer are fixed at bulk crystalline positions. To correctly describe the van der Waals interaction, we use a dispersion-corrected DFT method (optB88-vdW)^36^,^37^, which has been demonstrated to reliably describe 2D heterostructures. Figure 6(a,b) show the optimized geometrical structures obtained by examining various positions of the PbH relative to BN substrate. We find that the lattice mismatch is only about 0.05% and 0.41% for the BN-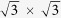 and BN-2 × 2 substrates, while the distance between adjacent layers are 3.697 Å and 3.578 Å, respectively. The binding energy is obtained to be 0.036 eV per unit cell, indicating a weak interaction between PbH and BN sheet. As expected from the band structure in Fig. 6(c,d), PbH on the BN sheet remains semiconducting. Bader charge analysis indicates that there is essentially no charge transfer between adjacent layers, thus the states around the Fermi level are dominantly contributed by PbH monolayer. In comparison to the pristine PbH, little difference is observed between them. Evidently, they are robust QSH insulators whose band inversion is not affected by the substrate.

In summary, based on first-principles calculations, we have predicted a new class of 2D QSH insulators in PbX monolayers with a giant-gap of 1.34 eV, allowing for viable applications at room temperature. This giant-gap opening is mainly due to the result of the strong SOC related to the *p*_*x,y*_ orbitals of Pb atoms at the Γ point, in sharp contrast to that consisting of the *p*_*z*_ orbital as in pristine group-IV ones. The topological characteristic of Pb-H monolayer is confirmed by the Z_2_ topological order due to *s-p*_*x,y*_ band inversion and an explicit demonstration of the topological edge states, while the strong SOC enhances nontrivial gaps greatly. Also, these systems maintain their nontrivial topological phase within the certain strain range, indicating their topology is robust against strain. We also propose high-dielectric-constant BN as an ideal substrate for the experimental realization of PbX, maintaining its nontrivial topology. These results represent a significant advance in the study of TIs and thus are expected to stimulate further work to synthesize, characterize and utilize PbX monolayers for fundamental exploration in spintronics.

## Methods

First-principles calculations were performed by using density functional theory (DFT) methods as implemented in the Vienna ab initio simulation (VASP)^38^,^39^ package. The projector-augmented-wave potential, the Perdew-Burke-Ernzerhof (PBE) exchange-correlation functional^40^,^41^, was used to to treat the ion-electron interactions. The energy cutoff of the plane waves was set to 500 eV with the energy precision of 10^−6 ^eV. The Brillouin zone was sampled by using a 21 × 21 × 1 Gamma-centered Monkhorst–Pack grid. The vacuum space was set to 20 Å to minimize artificial interactions between neighboring slabs. SOC was included by a second variational procedure on a fully self-consistent basis. With the optimized structures, the more sophisticated HSE06 hybrid functional^26^ was used to check the corresponding results of the systems. The phonon spectra were calculated using a supercell approach within the PHONON code^42^.

## Additional Information

**How to cite this article**: Zhao, H. *et al.* Unexpected Giant-Gap Quantum Spin Hall Insulator in Chemically Decorated Plumbene Monolayer. *Sci. Rep.* **6**, 20152; doi: 10.1038/srep20152 (2016).

## Supplementary Material

Supporting Information

## Figures and Tables

**Figure 1:**
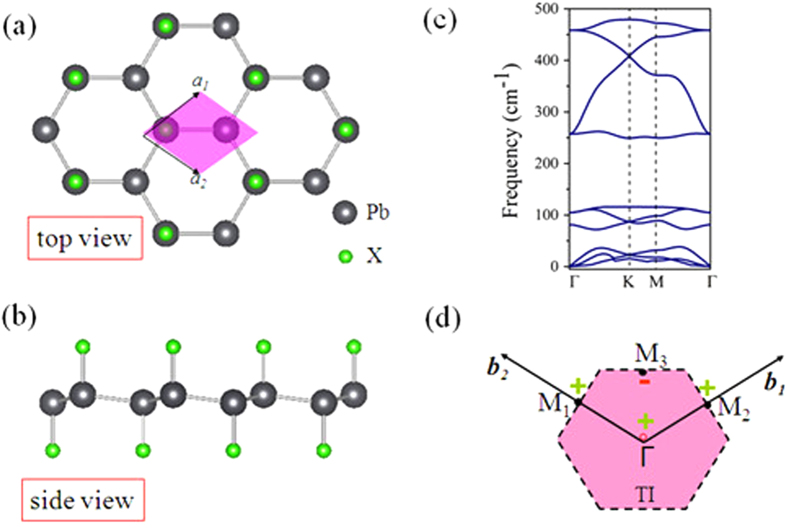
(**a**,**b**) Top and side views of the schematic structures of PbH monolayer. Black and green balls denote Pb and H atoms, respectively. Shadow area in (**a**) present the unit cell. (**c**) Phonon band dispersions, and (**d**) the area of Brilioun zone of PbH monolayer.

**Figure 2:**
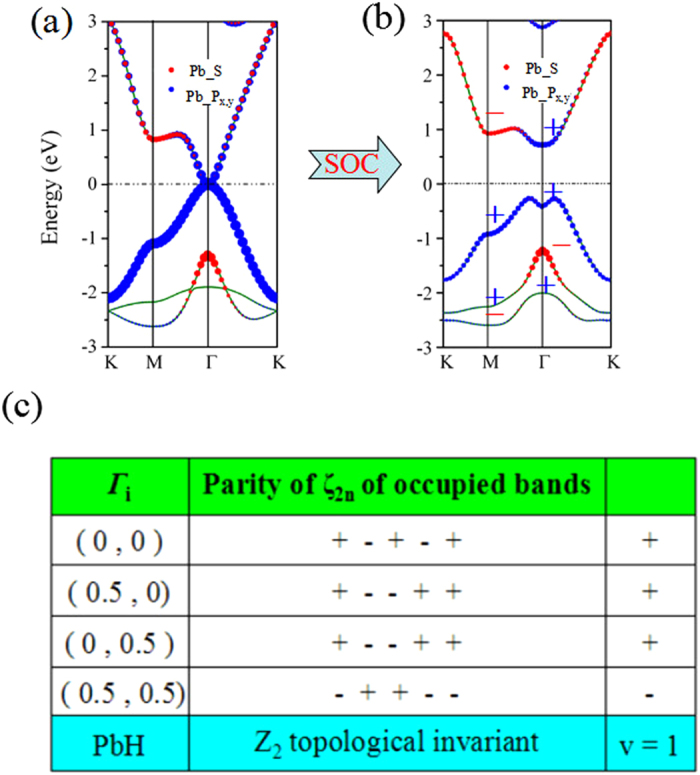
Band structures for PbH without SOC (**a**) and with SOC (**b**) with zooming in the energy dispersion near the Fermi level. The red circles and blue squares represent the weights of the Pb-*s* and Pb-*p*_*x,y*_ character, respectively. (**c**) Parities of occupied spin-degenerate bands at the TRIM Points for PbH. Here, we show the parities of 5 occupied spin-degenerate bands for PbH. Positive and negative signs denote even and odd parities, respectively.

**Figure 3:**
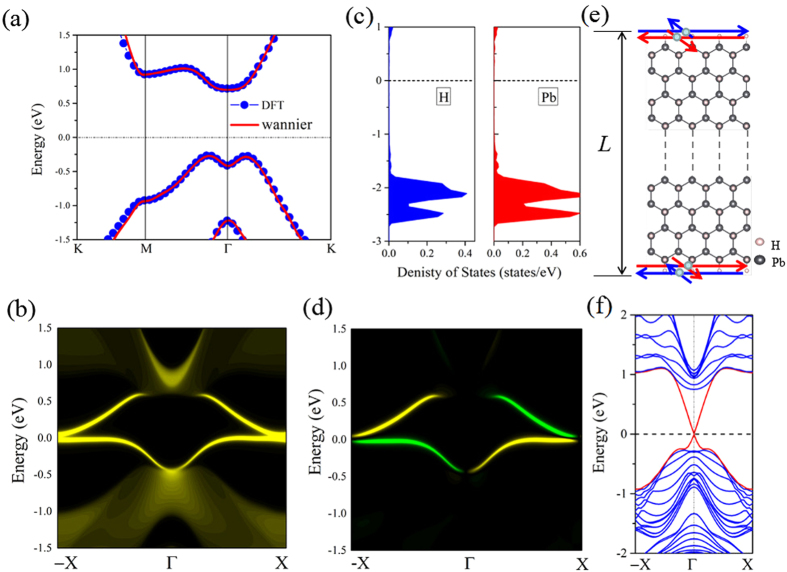
Electronic structures and its corresponding edge state of PbH. (**a**) Comparison of band structures for PbH calculated by DFT (red lines) and Wannier function method (blue circles). (**c**) Illustration of the partial DOS projected onto *p*_*z*_ orbital of Pb and the total DOS of H atom. The Fermi level is set to zero. (**b**,**d**) show the Dirac edge states, and edge spin polarization, respectively. The Fermi level is set to zero. (**e**,**f**) the model and spectrum of a finite slab of PbH. The red and blue horizontal arrows represent the spin-up and -down polarized currents in opposite direction.

**Figure 4:**
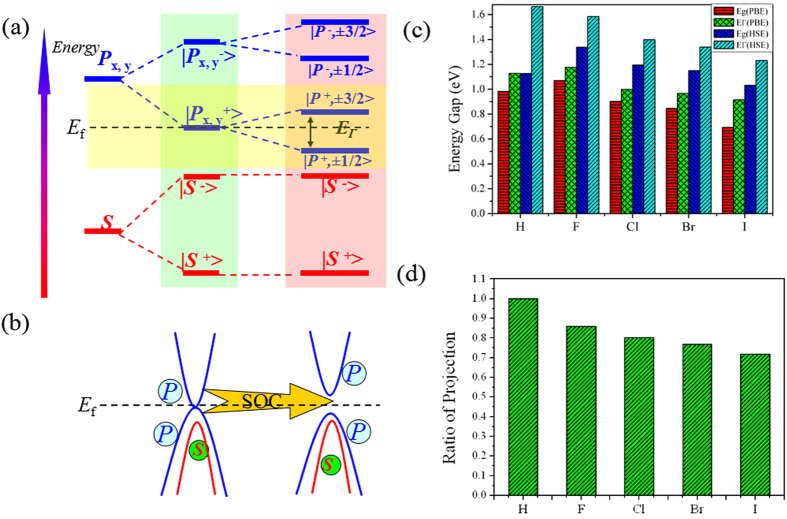
(**a**) The evolution of atomic *s* and *p*_x,y_ orbitals of PbH into band edges at Γ point is described as the crystal field splitting and SOC are switched in sequence. (**b**) Illustration of the effect of SOC on the inversion of bands around the Fermi level. (**c**) The calculated global band gap by PBE and HSE methods, and (**d**) the ratio of Pb-*p*_*x,y*_ component in the total orbital at Γ point near the Fermi level.

**Figure 5:**
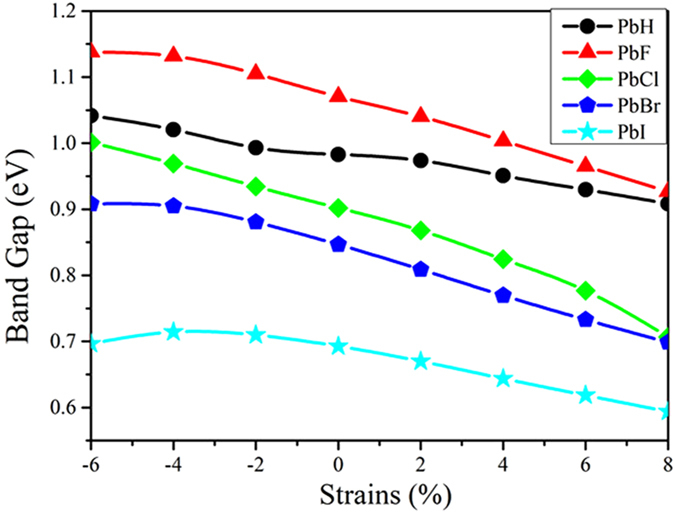
The calculated global band gap (*E*_g_) of PbX with SOC as a function of external strain.

**Figure 6:**
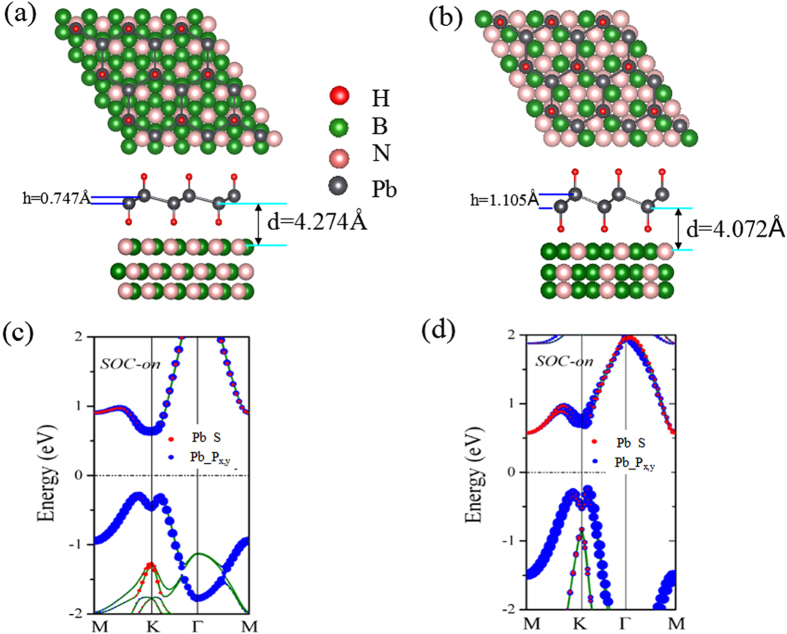
(**a**) Side and top views of the schematic illustration of the epitaxial growth PbH of large-gap QSH states on 2 × 2 BN substrate and (**b**) on 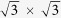
substrate. Orbital-resolved band structures with SOC based on DFT calculations. (**c**,**d**) notes the energy band of PbH on 2 × 2 and 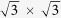 BN substrate with SOC. The Fermi level is set to zero.

**Table 1:** The present crystal parameters include the lattice constant a, Pb-Pb bond length d_1_, Pb-X bond length d_2_, buckling height ∆ (∆ is defined as the distance from the center of the upper to that of the lower Pb atoms), and the global band gap *E*_g_^PBE^ calculated with PBE, as well as the formation energy ∆E_f_.

monolayers	a(Å)	d_1_(Å)	d_2_(Å)	Δ(Å)	v_f_(10^5^m/s)	*E*_g_^PBE^(eV)	*ΔE*_*f*_ (eV/atom)	Z_2_
Pb	4.9257	2.9906	\	0.9256	\	0.421	\	0
PbH	5.0359	3.0098	1.8365	0.7781	2.5616	0.983	−1.89	1
PbF	5.1431	2.9902	2.0888	0.3518	1.2472	1.071	−2.82	1
PbCl	5.3017	3.1463	2.4934	0.4161	0.7215	0.902	−1.48	1
PbBr	5.3795	3.1424	2.6362	0.4779	0.6573	0.847	−1.32	1
PbI	5.4441	3.1346	2.8394	0.5528	0.4961	0.693	−0.86	1

The Fermi velocity is shown as v_f_, and topological invariant is defined as Z_2_.
