# Supplementary material for: Unexpected Giant-Gap Quantum Spin Hall Insulator in Chemically Decorated Plumbene Monolayer
Source: Sci Rep. 2016 Feb 2;6:20152. doi: 10.1038/srep20152 (PMC4735859; doi:10.1038/srep20152)
Supplement: Supporting Information [file srep20152-s1.pdf]

Supplementary Information

Unexpected Giant-Gap Quantum Spin Hall Insulator in Chemically Decorated Plumbene Monolayer

Hui Zhao,[a] Chang-wen Zhang*,[a] Wei-xiao Ji,[a] Run-wu Zhang,[a] Sheng-shi Li,[b] Shi-shen Yan,[b] Bao-min Zhang,[a] Ping Li,[a] and Pei-ji Wang [a]

[a] School of Physics and Technology, University of Jinan, Jinan, Shandong, 250022, People's Republic of China

[b] School of Physics, State Key laboratory of Crystal Materials, Shandong University, Jinan, Shandong, 250100, People's Republic of China

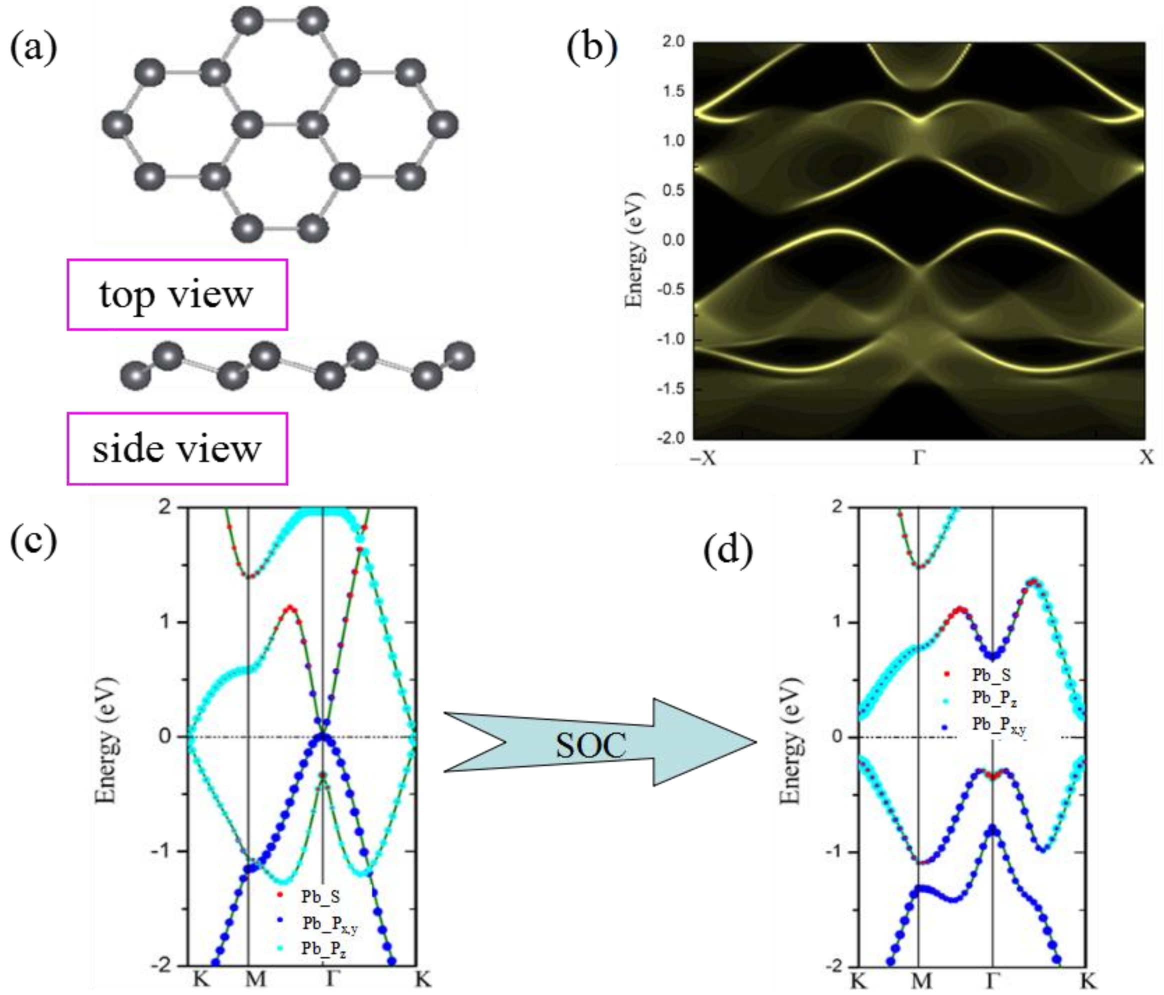

Fig. S1 The geometric structure and corresponding electronic properties of plumbene monolayer. Here, we call hexagonal Pb monolayer as 'plumbene'. The plumbene monolayer has the point group symmetry of D_{3d} with spatial inversion, exhibiting a bipartite honeycomb lattice with A and B sublattices. Different from sp^2 hybridization in graphene, the two sublattices have different heights, forming a buckled configuration. At the equilibrium state, the Pb-Pb bond length is about 2.89 Å and the height of buckling (h) is 1.64 Å with an angle of θ = 58.7°, as indicated in Fig. 1(a). On the other hand, the band structures of plumbene without and with SOC are present in 1(b) and 1(d). By projecting the bands onto different atomic orbitals, the energy spectrum near the Fermi level at K point mainly from Pb p_z orbitals, while at Γ point mainly comes from the $p_{x,y}$ orbitals. Especially, no Dirac point crosses linearly at the K point are observed in the absence of SOC, in sharp contrast to the cases of graphene and silicene. Although a band gap opens when SOC is turned on, the plumbene isn't a

QSH insulator, different from other goup IV counterpart. As expected in 1(b), there is no Dirac edge state in bulk band gap of plumbene monolayer. The edge Green's function of a semi-infinite Pb is constructed as shown in (c).

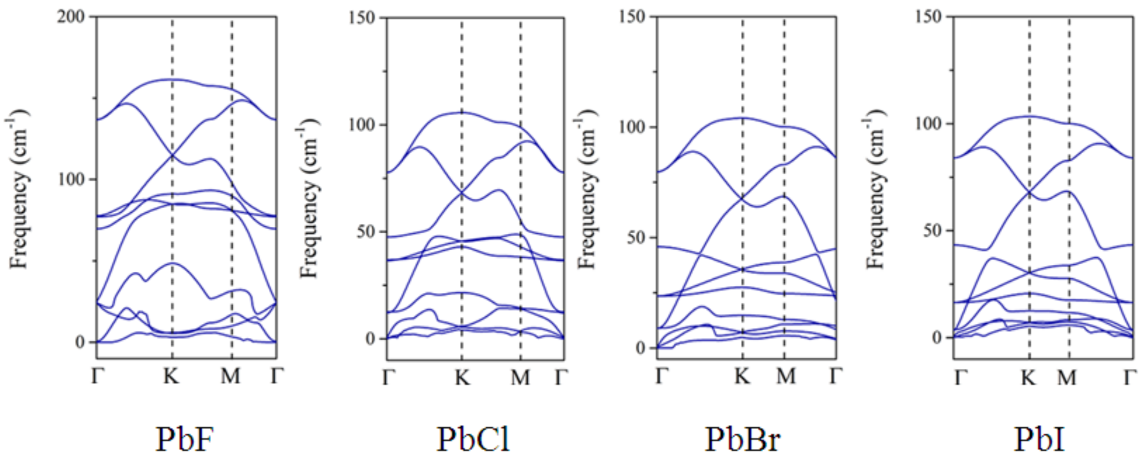

Fig. S2. The calculated Phonon band dispersions for PbF, PbCl, PbBr, and PbI monolayers, respectively.

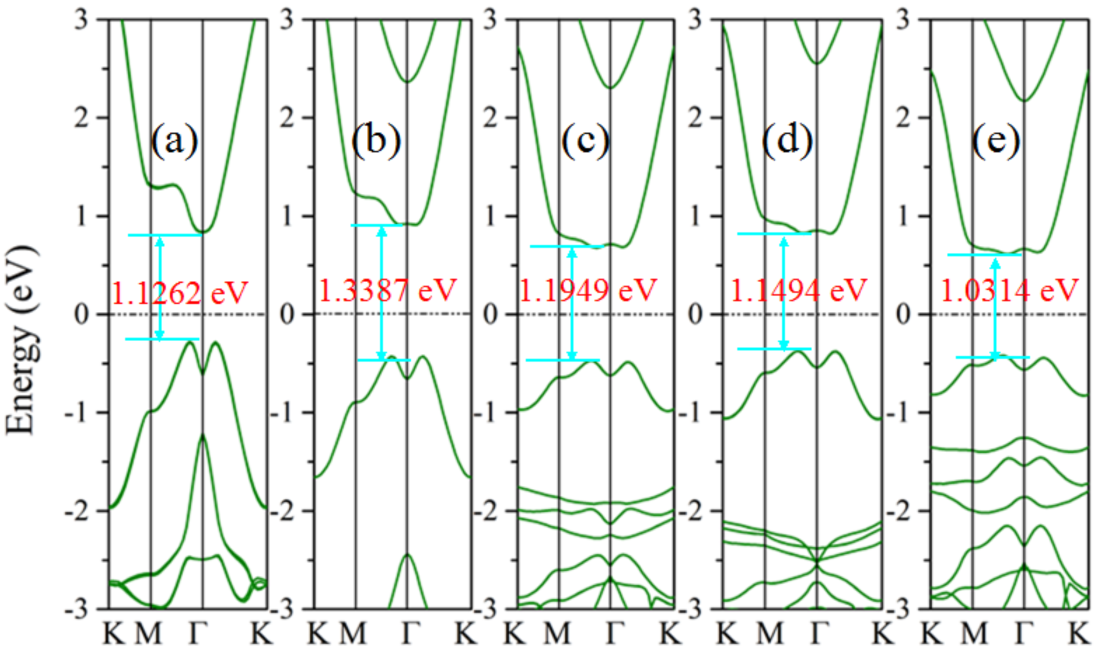

Fig. S3. The calculated band structure with HSE methods for (a) Pb-H, (b) Pb-F, (c) Pb-Cl, (d) Pb-Br, (e) Pb-I, respectively.

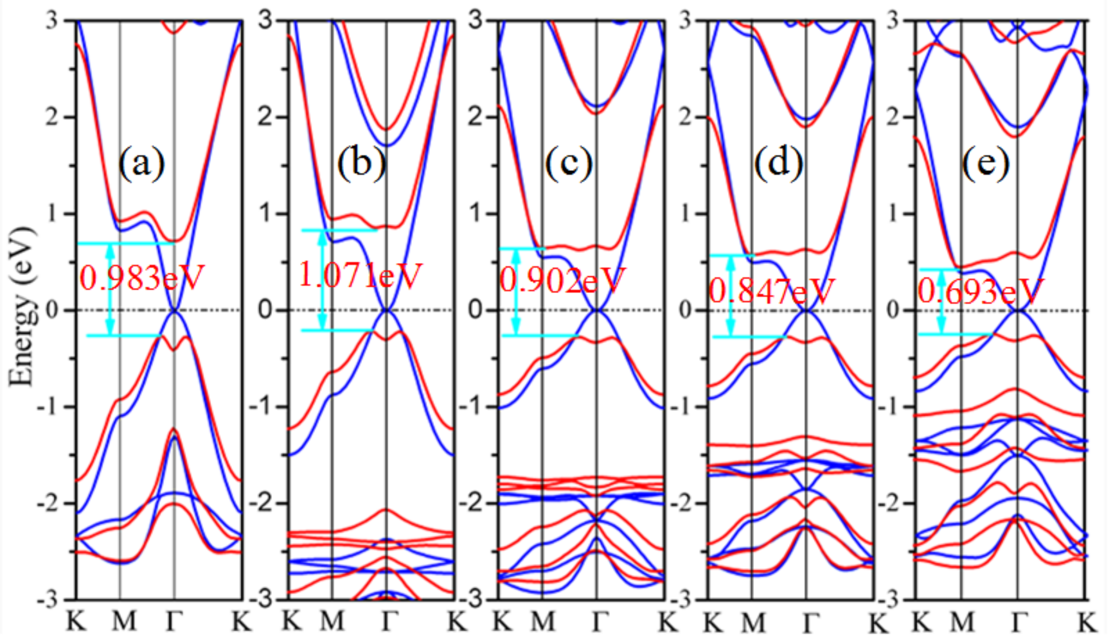

Fig. S4 The calculated band structures with PBE for (a) PbH, (b) PbF, (c) PbCl, (d) PbBr and (e) PbI monolayers, respectively.

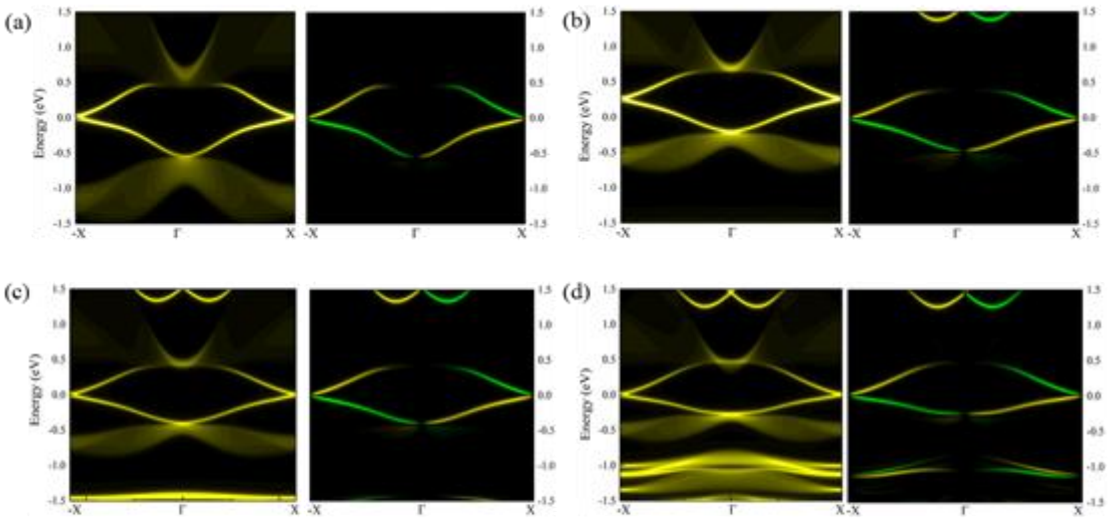

Fig. S5 Calculated local DOS of edge states for (a) PbF, (b) PbCl, (c) PbBr, and (d) PbI monolayers. The edge states are calculated on the edge of a semi-infinite plane. The yellow colors represent higher local density of states, while the black regions indicate 2D bulk energy bands and band gaps, respectively. And we also show the edge spin polarization with the yellow and green line.

Table S1 Parities of occupied spin-degenerate bands at the TRIM Points for PbX monolayers. Here, Positive and negative signs denote even and odd parities, respectively.

Γ i	**Parity of ζ2n of occupied bands**	**Product**
(0, 0)	+ - + - + + + - - - +	-
(0.5, 0)	+ - + - - + + - + - +	-
(0, 0.5)	+ - + - - + + - + - +	-
(0.5, 0.5)	- + - + + - - + - + -	+
PbF	Z_2 topological invariant	v = 1
(0, 0)	+ - + - + - + + - - +	-
(0.5, 0)	+ - + - - + + - + - +	-
(0, 0.5)	+ - + - - + + - + - +	-
(0.5, 0.5)	- + - + + - - + - + -	+
PbCl	Z_2 topological invariant	v = 1
(0, 0)	+ - + - + - + - + - +	-
(0.5, 0)	+ - + - - + + - + - +	-
(0, 0.5)	+ - + - - + + - + - +	-
(0.5, 0.5)	- + - + + - - + - + -	+
PbBr	Z_2 topological invariant	v = 1
(0, 0)	+ - + - - + + - + - +	-
(0.5, 0)	+ - + - - + + - + - +	-
(0, 0.5)	+ - + - - + + - + - +	-
(0.5, 0.5)	- + - + + - - + - + -	+
PbI	Z_2 topological invariant	v = 1
